# Reluctance to Use a Psycho-Oncology Mobile App Among Patients With Primary Breast Cancer: Retrospective Cross-Sectional Survey

**DOI:** 10.2196/71412

**Published:** 2026-02-13

**Authors:** Marta Pawełczak-Szastok, Anna Syska-Bielak, Aleksandra Krzywon, Michał Jarząb

**Affiliations:** 1 Department of Bone Marrow Transplantation and Oncohematology, Maria Sklodowska-Curie National Research Institute of Oncology, Wybrzeże Armii Krajowej Street 15, Gliwice, 44-100, Poland, 48 32 278 85 69; 2 Center for Diagnosis and Treatment of Breast Diseases, Maria Sklodowska-Curie National Research Institute of Oncology, Gliwice, Poland; 3 Department of Biostatistics and Bioinformatics, Maria Skłodowska-Curie National Research Institute of Oncology, Gliwice, Poland

**Keywords:** eHealth, eHealth barriers, breast cancer, psychoemotional functioning, psycho-oncology, refusal to participate, mobile app, clinical trial

## Abstract

**Background:**

eHealth is an increasingly used method of health care in the field of psycho-oncology. While many reports highlight the positive impact of psychological eHealth tools, some patients refuse to use them.

**Objective:**

This study aimed to expand knowledge of the motivation and psychoemotional functioning of patients who consciously refuse to use eHealth technology in the form of a mobile psycho-oncology app offered as part of a clinical trial. To our knowledge, this is the first study to address this topic.

**Methods:**

A retrospective cross-sectional study was conducted between December 2022 and February 2023 to investigate the reasons why 56 patients with breast cancer refused to use the psycho-oncology mobile app offered as part of a clinical trial by the Breast Cancer Unit. The primary aim of the study was to analyze patients’ self-reported reasons for not engaging with the app, while also exploring their psychoemotional functioning, including stress levels (measured using the distress thermometer), personality traits (measured using the Ten-Item Personality Inventory), coping strategies (measured using the Coping Orientation to Problems Experienced Questionnaire), and Self-efficacy (measured using the General Self-Efficacy Scale). Participants in this study declined the app intervention but agreed to participate in this separate observational study, indicating that their refusal was related to the app itself rather than to participation in clinical research in general.

**Results:**

The patients experienced a clinically meaningful elevation in stress levels (mean 5, SD 2.1 points) and Self-efficacy (mean 32.1, SD 5.1 points). Among 5 dimensions of personality traits, patients scored highest in Agreeableness (mean 6.5, SD 0.8 stens) and Conscientiousness (mean 6.4, SD 0.9) and lowest in Neuroticism (mean 3.4, SD 1.8) (other dimensions: Extraversion [mean 5.8, SD 1.6 stens] and Openness to Experiences [mean 4.4, SD 1.5 stens]). In terms of coping with stress, patients most frequently used the strategies of Active Coping (mean 2.6, SD 0.5 points), Acceptance (mean 2.6, SD 0.6 points), and Seeking Emotional Support (mean 2.6, SD 0.6 points), and least frequently used the strategies of Psychoactive Substance Use (mean 0.2, SD 0.6 points) and Restraint (mean 0.5, SD 0.7 points). Patient responses regarding refusal to participate in app testing were divided into four categories: (1) Focus on Life Outside the Disease, (2) Focus on Disease and Treatment, (3) Denial Mechanism, and (4) Technical Issues. Statistically significant differences were found between the groups. The Focus on Life Outside the Disease group of patients had higher levels of Self-efficacy, lower Neuroticism, and more frequent use of the Positive reevaluation strategy compared to the other groups.

**Conclusions:**

Our patients’ decision not to use the eHealth psycho-oncology app was mainly influenced by characteristics suggesting better emotional coping with the disease and treatment. These factors were significantly more influential than other factors studied, particularly those related to technology. Assessing reasons for opting out of eHealth and associated psychomotional functioning may be important for improving patients’ adoption of eHealth solutions.

## Introduction

Electronic communication–based tools (eHealth) are a method of providing health care services using web-based technologies [1]. eHealth has been developing for more than a decade [2-5]. Originally designed to expand access to health care through remote contact and reduce costs, during the COVID-19 pandemic, these solutions became increasingly common and entered the mainstream of care due to the requirements of social isolation [6]. The solutions developed during this period have proved beneficial to the health care system in terms of time, premises, and economy. Researchers emphasize widespread availability, improved health monitoring among the public, and increased awareness of healthy lifestyles and ways to prevent diseases and other undesirable psychological conditions [2,3]. Many reports indicate the positive impact of psychological eHealth tools, highlighting a number of their advantages [7-11]. eHealth solutions are also increasingly being used in specific areas of health care, such as psycho-oncology [2,12]. Their goal is to improve psychological well-being both during and after oncological illness. Most of these tools are based on the tenets of cognitive-behavioral therapy and are constantly being developed [13].

The number of publications regarding barriers to the use of eHealth technologies is lower than that outlining its advantages [14]. Most emphasize significant barriers, including inability to access the technology, lack of time for patients and clinicians, and lack of patient motivation or need to use it [14-16]. However, fears associated with the risk to privacy for health-related data, lack of trust in algorithm-based approaches, and the absence of optimal, safe, and trustworthy channels for the dissemination of distant eHealth tools are also vital potential limitations.

Few publications address the barriers associated with using eHealth solutions in the field of psycho-oncology, and even fewer provide evidence specific to cancer types [2]. In a comprehensive systematic review of eHealth technologies for breast cancer supportive care, Gyawali et al [2] identified only 2 studies reporting information on barriers to the use of eHealth interventions. These studies mention 2 groups of barriers. The first group is related to the failure to meet the necessary conditions for effective use of eHealth, including awareness of, openness to, and trust in eHealth among both patients and staff, digital competence, and a clear legal framework. The second group of barriers is mainly related to the disadvantages reported by patients during use, such as technical problems, mismatch between content and stage of treatment, lack of certain information, and discomfort with the lack of face-to-face contact.

In another recent review by Horn et al [17] on breast cancer survivors, 8 trials were identified that reported reasons for dropouts. The most frequent reason was time constraints, followed by unmet expectations of participants, technical problems, and physical and psychological health. In addition, researchers note the lack of uniform assessment scales for the usefulness of eHealth tools, and existing assessments mainly focus on technical aspects [18]. They also highlight that professionals have their own barriers to using eHealth [19].

Most studies on barriers to the use of eHealth in psycho-oncology have been conducted among patients who have used such solutions, reporting their perceived advantages and disadvantages [2,17]. We found only 1 survey that explicitly addressed patients who refuse to use such solutions [20]. This group of patients seems particularly interesting because of the growing demand for eHealth and the prospect that, in the near future, some interventions will only be possible using eHealth. Gupta et al [20] described only challenges in the recruitment process. Our goal was to expand knowledge of the motivation and psychoemotional functioning of patients who consciously refuse to use eHealth technology in the form of a mobile psycho-oncology app offered as part of a clinical trial. Specifically, we examined reasons for refusal and assessed patients’ stress levels, Self-efficacy, personality traits, and coping strategies. Importantly, all participants included in this study declined the mobile app intervention but agreed to participate in a separate observational study related to psychoemotional functioning. This distinction indicates that their refusal was specific to the app itself rather than to a general reluctance to participate in clinical research. Therefore, this study aimed to explore and categorize the reasons for refusal of a mobile psycho-oncology app among patients with primary breast cancer and to examine the psychoemotional functioning associated with different refusal categories. To the best of our knowledge, this is the first study of its kind.

## Methods

### Materials and Procedure

We conducted a retrospective study at the Breast Cancer Unit of the National Research Institute of Oncology in Gliwice, Poland, between February 2022 and November 2022. During this period, 138 patients with primary early-stage breast cancer at the beginning of the treatment process were offered participation in a clinical trial involving the use of a psycho-oncology mobile phone app. The clinical trial ran from February 2022 to May 2023. This clinical investigation concerned a medical device; therefore, in accordance with regulatory requirements for medical device trials, it was not assigned a clinical trial registration number (eg, European Union Drug Regulating Authorities Clinical Trials database). The app aimed to support the adaptation to the diagnosis and treatment of cancer based on cognitive-behavioral therapy. It comprised 22 visually appealing lessons, practical exercises (breathing exercises, imagery-based exercises, values-based activities, mindfulness exercises, and gratitude practice), emotion monitoring, and practical information and tips. The app was introduced to patients through a short video and was provided at no cost to those who agreed to join the clinical trial to evaluate its effectiveness. The details of the intervention are described elsewhere [21], and the final trial analysis is pending.

**Figure 1.**
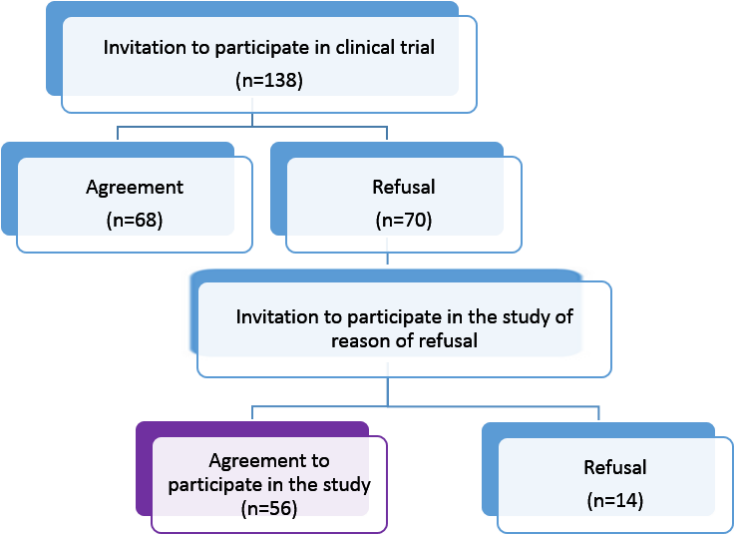
Flowchart of study selection for evaluating the mobile psycho-oncology app among patients with primary breast cancer, Gliwice, Poland (2022-2023).

This report specifically focuses on patients who declined to use the mobile app within the clinical trial. Of the 138 female patients who were offered participation in the trial, 70 (51%) refused to use the app. A subsequent study involving individuals who declined to use the app was conducted from December 2022 to February 2023. Of the 70 patients who declined, 56 agreed to participate in this study. Notably, among the remaining 14 patients who declined to participate, 6 did not attend the study appointment, and 5 provided no reason for their refusal other than unwillingness to be tested. These individuals may be considered potentially reluctant to participate in research in general. In contrast, 56 patients agreed to participate in our study, which suggests that their previous refusal was not driven by a general aversion to research but rather by specific concerns or disinterest regarding the mobile app intervention. In addition, 3 patients declined participation because of significant deterioration in health and severe pain symptoms, making it impossible to engage with a psychologist. The study recruitment and selection process is provided in Figure 1. The reporting of this observational study follows the STROBE (Strengthening the Reporting of Observational Studies in Epidemiology) guidelines.

### Measures

#### Demographic and Clinical Factors

Each participant answered survey questions regarding demographic issues, including age, marital status, and parental status. We reviewed patients’ electronic medical records to obtain data regarding cancer diagnosis, treatment stage, date of diagnosis, and initial level of distress measured during the initial psychological interview conducted routinely for all patients at the local Breast Unit.

#### Reasons for Refusal

Reasons were collected using an open-ended question specifically constructed and designed for this study. Patients were asked: “Please indicate the main reason why you decided not to use the psycho-oncology mobile app offered as part of the study.” Participants provided their responses in writing during an individual session conducted in the presence of a clinical psychologist, who was available to clarify instructions and provide support if needed. The assessment session took place during a subsequent hospital visit, separate from the encounter in which the patient initially declined participation in the mobile app trial. Two clinical psychologists independently coded the responses, identifying a total of 10 categories of refusal reasons. The categorization followed a data-driven process: each psychologist reviewed the answers separately and generated preliminary codes reflecting the core meaning of participants’ statements. Discrepancies were resolved through consensus discussion. These initial codes were then grouped into broader thematic categories based on conceptual similarity and clinical relevance. Where applicable, the process was informed by previous literature, particularly Gupta et al [20], who described barriers to the adoption of eHealth interventions among patients with breast cancer. While most of our categories corresponded to domains identified in earlier research, one clinically important category—reluctance to recall disease-related content—emerged uniquely from our data and is discussed further in the “Discussion” section. Importantly, the responses captured in this process referred specifically to the patients’ decision to decline the mobile app, not to research participation in general. This combined approach ensured that the final categorization was both empirically grounded and clinically meaningful. The thematic categories identified through this process are summarized in Textbox 1, which presents the clinically relevant reasons for declining the psycho-oncological app.

Textbox 1. **Reasons for refusal to use the psycho-oncological app within the clinical trial, Gliwice, Poland (2022‐2023).**

**Lack of time—childcare responsibilities:**

- Participants reported limited time due to childcare responsibilities, such as having a small child and being unable to find time for additional activities.

**Lack of time—difficult family situation:**

- Some respondents were engaged in caring for a disabled family member, including a disabled mother, child, or spouse.

**Lack of time—demanding job:**

- Several participants indicated being highly absorbed in professional work, describing their jobs as very responsible and demanding, leaving no available time.

**Lack of time—frequency of medical appointments:**

- Frequent medical examinations, consultations, and hospitalizations resulted in a lack of time for further commitments.

**Poor physical and mental well-being:**

- Respondents described mental exhaustion and poor physical condition, limiting their ability to focus on additional tasks.

**Reluctance to recall disease-related content:**

- Some participants did not wish to think about their illness at home and preferred to avoid disease-related content.

**No mental need, feeling good:**

- Several patients reported not feeling a need for psychological support or that the diagnosis did not significantly affect their well-being, reducing interest in a psycho-oncology app.

**Reluctance toward new technologies:**

- Some respondents expressed reluctance to use new technologies and a lack of interest in mobile apps.

**Refusal to phone contact:**

- Reasons included not having a private mobile phone or being unable to install non–work-related apps.

**Other reasons:**

- This category included various individual reasons, such as already receiving sufficient support through long-term psychotherapeutic treatment.

The process involved categorizing the responses into overarching categories based on the focus of the patients’ concerns and reasons for refusal. The essential criterion for assignment to the various categories was the primary reason behind the refusal, as identified by 2 independently working psychologists. None of the responses suggested a refusal motivated by distrust or avoidance of research participation per se. This further supports the interpretation that these patients’ refusals were app-specific rather than reflecting a general reluctance to engage in studies. This analysis allowed for the combination of basic categories into 5 overarching categories, providing a comprehensive understanding of the patients’ perspectives and motivations for refusal: (1) Focus on Life Outside the Disease, (2) Focus on Disease and Treatment, (3) use of a Denial Mechanism in relation to content related to illness and treatment, (4) Technical Issues, and (5) Other Reasons. The process of categorizing responses with examples of patients’ statements is provided in Table S1 in Multimedia Appendix 1.

The distress thermometer, a self-report measure of psychological distress, with an 11-point range from “no distress” (0) to “extreme distress” (10), resembling a thermometer [22,23], was used. Respondents were instructed to circle the number (0‐10) that best describes their level of distress on the day of measurement. The validated Polish version was used; according to the validation results, a threshold of 5 points provides the greatest balance of sensitivity and specificity in selecting patients with severe distress.

The General Self-Efficacy Scale (GSES) by Schwarzer [24] was used to evaluate Self-efficacy beliefs. Self-efficacy is defined as the ability or level of confidence in performing specific behaviors. The GSES consists of 10 questions, rated on a 4-point Likert scale ranging from 1 (“strongly disagree”) to 4 (“strongly agree”). The total score ranges from 10 to 40 points, with higher scores indicating a stronger sense of self-efficacy. Raw scores can be converted into standardized sten scores. The scale has demonstrated good reliability and measurement validity. The GSES has been translated into Polish by Juczyński [24].

The Ten-Item Personality Inventory (TIPI), a concise tool designed to evaluate the 5-factor personality model established by Costa and McCrae, was applied in the study [25]. TIPI was specifically developed to offer a brief (1 minute) assessment option in situations where using more comprehensive instruments would be unfeasible. The model encompasses Extraversion, Agreeableness, Conscientiousness, Emotional stability (or Neuroticism), and Openness to Experiences (10 items with five 2-item subscales). Respondents assigned a Likert score where 1 indicates “disagree strongly,” and 7 indicates “agree strongly,” with raw scores transformed into standardized sten scores. Correlations between the Polish version of TIPI and NEO-FFI confirm the convergent and discriminant validity of TIPI.

The Coping Orientation to Problems Experienced Questionnaire (Mini-COPE) was used to assess stress coping. The Mini-COPE, developed by Carver et al [26] and adapted into Polish by Juczyński and Ogińska-Bulik [27], comprises 28 items with 4 possible responses: “I haven’t been doing this at all” (0 points), “A little bit” (1 point), “A medium amount” (2 points), and “I’ve been doing this a lot” (3 points). Two items constitute one strategy, resulting in a total of 14 stress-coping strategies divided into 4 factors: Active Coping (comprising 3 strategies: Active Coping, Planning, and Positive Reinterpretation); Seeking Support (comprising 2 strategies: Seeking Emotional Support and Seeking Instrumental Support); Helplessness (comprising 3 strategies: Psychoactive Substance Use, Restraint, and Self-Blame); Avoidant Coping (comprising 3 strategies: Dealing with something else, Denial, and Venting). In addition, there are 3 strategies not belonging to any of the above-mentioned factors: Religion, Acceptance, and Humor. All strategies can be categorized into Active (effective) strategies and passive (ineffective) strategies. Active strategies include Active Coping, Seeking Support, Religion, Acceptance, and Humor, while passive strategies encompass Helplessness and Avoidant Coping. The questionnaire’s split-half reliability was 0.86, and the internal consistency for most scales was assessed as satisfactory.

### Statistical Analysis

Categorical variables were summarized as frequencies and percentages. Continuous data were summarized as median values with IQR 25%-75%. For continuous variables, comparisons between more than 2 groups were performed by the Kruskal-Wallis H test and post hoc Dunn test with Benjamini-Hochberg *P* value correction. The Spearman correlation coefficient was assessed to examine correlations between variables. The classification of correlations used to evaluate the analyzed results was as follows: 0.0≤|*r*|<0.1 negligible correlation; 0.1≤|*r*|≤0.39, weak correlation; 0.4≤|*r*|≤0.69, moderate correlation; 0.7≤|*r*|≤0.89, strong correlation; and 0.9≤*r*≤1, very strong correlation [28]. Hierarchical clustering was based on Euclidean distance. Colors were scaled per column. A 2-sided *P*<.05 was considered statistically significant. Computations were performed in the R environment for statistical computing (version 4.0.1; R Foundation for Statistical Computing), released on June 6, 2020 [29].

### Ethical Considerations

The study was approved by the Ethics Committee of the Maria Sklodowska-Curie National Research Institute of Oncology, Gliwice Branch (approval number KB/430-66/23). All procedures involving human participants were conducted in accordance with applicable ethical standards. Informed consent was obtained from all participants. Participant data were anonymized before analysis, and all personal information was handled confidentially in accordance with institutional data protection policies. No compensation was provided to participants for their involvement in the study.

## Results

### Participants

In the study group (N=56), all patients were diagnosed with primary early breast cancer (primary epithelial malignant tumors of the breast, at clinical stages I–III, eligible for treatment with curative intent, and with an Eastern Cooperative Oncology Group performance status of ≤2). The time since diagnosis ranged from 4 to 11 months, with most of the women undergoing preoperative systemic treatment (34/56, 60%). The age range was 32‐67 years, with a mean age of 50 (SD 9) years. The majority of patients were married (40/56, 71%) and had at least one child (50/56, 89%). Demographic and clinical data are provided in Table 1.

**Table 1.** Sociodemographic and clinical characteristics of patients with primary breast cancer who refused to test the mobile psycho-oncology app within the clinical trial, Gliwice, Poland (2022‐2023; N=56).

Characteristic	Value, n (%)	Value, mean (SD)	Value, median (IQR)
Age (years)	—^a^	50 (9)	48 (44-57)
Relationship status			
Married	40 (71)	—^a^	—^a^
Single	6 (11)	—^a^	—^a^
Divorced	7 (12)	—^a^	—^a^
Widowed	3 (5)	—^a^	—^a^
Children			
Yes	50 (89)	—^a^	—^a^
No	6 (11)	—^a^	—^a^
Number of children			
1	15 (27)	—^a^	—^a^
2	28 (50)	—^a^	—^a^
3	6 (11)	—^a^	—^a^
4	1 (2)	—^a^	—^a^
Average age of children	—^a^	21 (12)	23 (10-31)
Treatment stage			
During preoperative systemic treatment	35 (64)	—^a^	—^a^
After systemic therapy	20 (37)	—^a^	—^a^

a Not applicable.

### Reasons for Refusal to Participate in Testing the App

A total of 138 women were offered participation in a clinical trial testing a psycho-oncology mobile app. Of these, 70 (51%) patients declined to use the app and were subsequently invited to participate in this observational study, and 56 of these 70 patients (40.1%, 56/138) agreed to participate in this study and provided open-ended responses regarding their reasons for refusing the intervention. As detailed in the “Methods” section, the responses were coded and grouped into categories. The most frequently given reason was lack of time due to numerous medical appointments and examinations related to cancer diagnosis and treatment (13/56, 23%). The second most frequent answer (8/56, 14%) was lack of mental need or mental well-being. Other reasons included poor physical and mental well-being (7/56, 13%), reluctance to recall content related to illness (7/56, 13%), lack of time due to difficult family situation (6/56, 11%), reluctance toward new technologies (5/56, 9%), lack of time due to caring for a young child (4/56, 7%), lack of time due to demanding professional work (3/56, 5%), and lack of private telephone (2/56, 4%). One patient (1/56, 2%) had been in psychotherapeutic contact for many years and found it sufficient support for psycho-oncological issues as well. An analysis of the answers given by 2 independent psychologists working independently of each other made it possible to combine the above 10 answers into 5 main categories, of which the most numerous was “Focus on Disease and Treatment” (20/56, 36%). Less common were the reasons associated with denial mechanisms in relation to content associated with illness and treatment (15/56, 27%), life outside the disease (13/56, 23%), Technical Issues (7/56, 13%), and Other Reasons (1/56, 2%) (Table 2).

**Table 2.** Reasons for refusal to use the mobile psycho-oncology app in the clinical trial among patients with primary breast cancer, Gliwice, Poland (2022‐2023; N=56).

Overarching categories of reasons and basic reasons	Value, n (%)
Focus on life outside the disease	13 (23)
Lack of time—childcare responsibilities	4 (7)
Lack of time—difficult family situation	6 (11)
Lack of time—demanding job	3 (5)
Focus on the disease and treatment	20 (36)
Lack of time—frequency of medical appointments	13 (23)
Poor physical and mental well-being	7 (13)
Denial reaction	15 (27)
Reluctance to recall disease-related content	7 (13)
No mental need, feeling good	8 (14)
Technical issues	7 (13)
Reluctance toward new technologies	5 (9)
Refusal to phone contact	2 (4)
Other reasons	1 (2)

### Psychological Outcomes

There were no missing values in the psychological assessments. The mean score in the patients’ stress level measured with the distress thermometer was 5.0 (SD 2.1). The average level of distress in the whole group was moderate, close to the cut-off point according to Polish standards [23]. When analyzing the distribution of scores, we found that 63% of patients exceeded the cut-off point and 26% had a score of 7 or higher, indicating a significant escalation of distress. This means that at least a quarter of the patients in our study indicated a clinically significant escalation of distress. The mean score of generalized Self-efficacy measured with GSES was 32.1 (SD 5; stens: means 7.4, SD 1.9). Analysis of the 5 dimensions of personality measured using the TIPI questionnaire in patients refusing psycho-oncological eHealth support indicated the highest scores in the Conscientiousness dimension (mean 6.4, SD 0.9). The lowest score was obtained in the Neuroticism dimension (mean 3.4, SD 1.8). Mean scores for other personality traits were average: Extraversion (mean 5.8, SD 1.6), Agreeableness (mean 6.5, SD 0.8), and Openness to Experiences (mean 4.4, SD 1.5) (Table 3).

**Table 3.** Results of participants’ stress level (distress thermometer [DT]), Generalized Self-Efficacy Scale (GSES), and personality trait characteristic (Ten-Item Personality Inventory [TIPI]) among patients with primary breast cancer who declined participation in the mobile psycho-oncology app clinical trial, Gliwice, Poland (2022‐2023; N=56).

Questionnaire and variable	Value, mean (SD)	Value, median (IQR)
DT^a^		
Level of stress	5.0 (2.1)	5.0 (3.0-6.8)
TIPI^b^		
Extraversion	5.8 (1.6)	6.5 (5.4-7.0)
Agreeableness	6.5 (0.8)	6.8 (6.4-7.0)
Conscientiousness	6.4 (0.9)	7.0 (6.0-7.0)
Neuroticism	3.4 (1.8)	3.0 (2.0-4.5)
Openness to experience	4.4 (1.5)	4.5 (4.0-5.5)
GSES^c^		
Self-efficacy	32.1 (5.1)	32.5 (29.0-36.0)
Stens	7.4 (1.9)	7.5 (6.0-9.0)

a DT: distress thermometer.

b TIPI: Ten-Item Personality Inventory.

c GSES: General Self-Efficacy Scale.

The results in the Mini-COPE showed that patients most often used the strategies of Active Coping (mean 2.6, SD 0.5), Acceptance (mean 2.6, SD 0.6), and Seeking Emotional Support (mean 2.6, SD 0.6). Also relatively common were the strategies of Planning (mean 2.4, SD 0.6), Positive Reinterpretation (mean 2.3, SD 0.7), Seeking Instrumental Support (mean 2.2, SD 0.8), and Dealing with something else (mean 2.1, SD 0.8). The least used strategies were Psychoactive Substance Use (mean 0.2, SD 0.6), Restraint (mean 0.5, SD 0.7), and Sense of humor (mean 0.8, SD 0.7) (Table 4).

**Table 4.** Coping strategies (Coping Orientation to Problems Experienced Questionnaire [Mini-COPE]) among patients with primary breast cancer who declined to test the mobile psycho-oncology app within the clinical trial, Gliwice, Poland (2022‐2023; N=56).

Type, dimension, and name of stress coping strategies	Value, mean (SD)	Value, median (IQR)
Active strategies mean 2.1 (SD 0.94); median 2.5 (IQR 1.5-3.0)		
Active coping	2.4 (0.65)	2.5 (2.0-3.0)
Active coping	2.6 (0.5)	3.0 (2.5-3.0)
Planning	2.4 (0.6)	2.5 (2.0-3.0)
Positive reinterpretation	2.3 (0.7)	2.5 (1.5-3.0)
Acceptance	2.6 (0.6)	3.0 (2.5-3.0)
Sense of humor	0.8 (0.7)	1.0 (0.0-1.5)
Turning to religion	1.6 (1.2)	2.0 (0.5-3.0)
Seeking support	2.4 (0.72)	2.5 (2.0-3.0)
Seeking emotional support	2.6 (0.6)	3.0 (2.0-3.0)
Seeking instrumental support	2.2 (0.8)	2.5 (1.5-3.0)
Passive strategies mean 1.1 (SD 1.0); median 1 (IQR 0.0-2.0)		
Avoidance	1.5 (0.93)	1.5 (1.0 -2.0)
Dealing with something else	2.1 (0.8)	2.0 (1.5-3.0)
Denial	1.0 (0.9)	1.0 (0.0-1.5)
Venting	1.5 (0.8)	1.5 (1.0-2.0)
Helplessness	0.6 (0.86)	0.0 (0.0 -1.0)
Psychoactive substance use	0.2 (0.6)	0.0 (0.0-0.0)
Restraint	0.5 (0.7)	0.0 (0.0-1.0)
Self-blame	1.1 (1.0)	1.0 (0.0-2.0)

**Figure 2.**
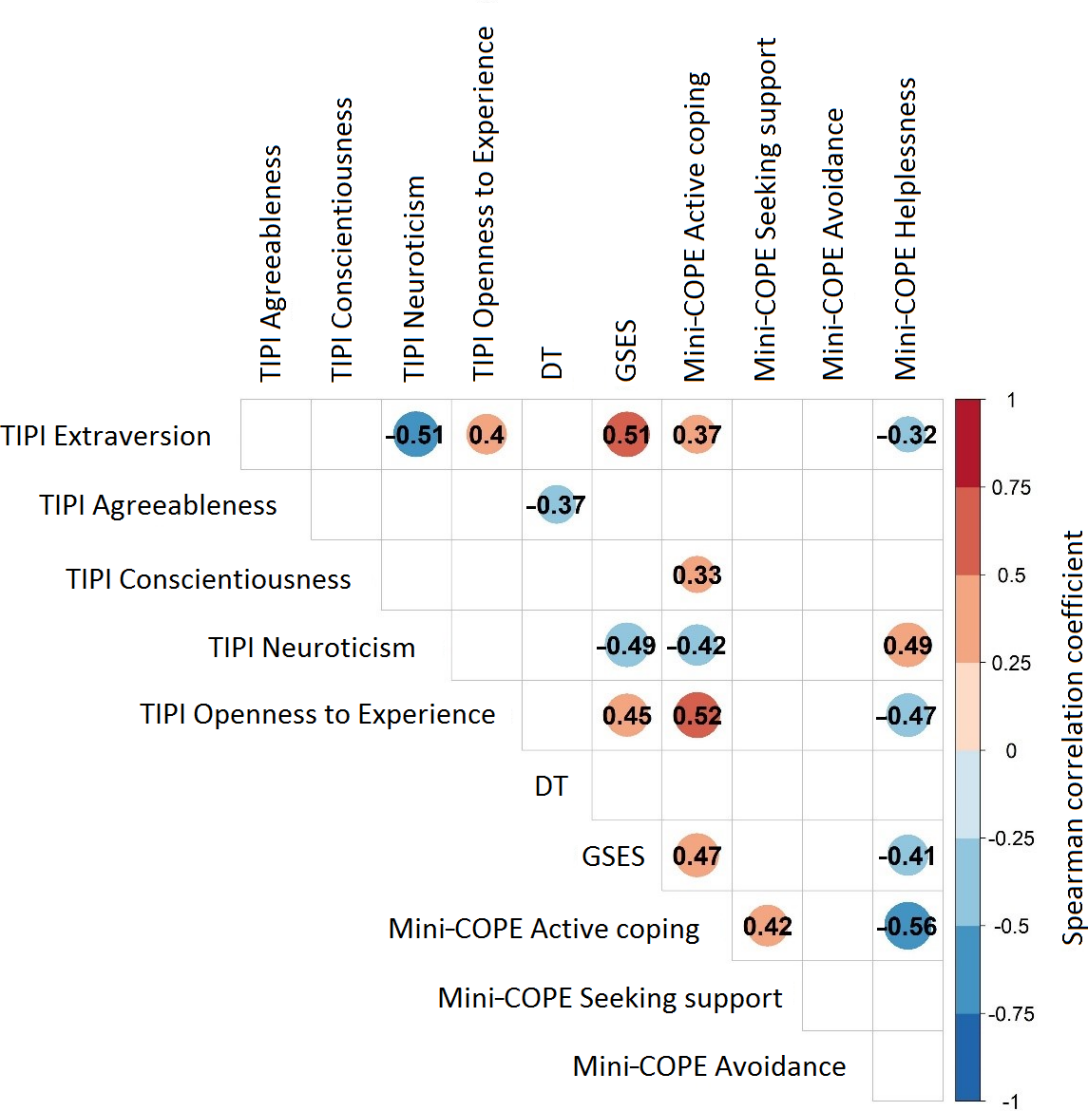
Spearman correlation coefficient between stress level (distress thermometer [DT]), Generalized Self-Efficacy Scale (GSES), personality trait characteristics (Ten-Item Personality Inventory [TIPI]), and stress coping strategies (Coping Orientation to Problems Experienced Questionnaire [Mini-COPE]) among patients with primary breast cancer who declined to use the mobile psycho-oncology app within the clinical trial, Gliwice, Poland (2022-2023). A blank space indicates an insignificant correlation (*P*≥.05).

In the next step, correlations between individual variables of psychoemotional functioning were analyzed to identify possible personality profiles of patients in our study who refused to test the eHealth app. We observed moderate positive correlations between Extraversion and Self-Efficacy (*r*=0.51; *P*<.001), Helplessness and Neuroticism (*r*=0.49; *P*<.001), Self-Efficacy and Active Coping (*r*=0.47; *P*<.001), Openness to Experience and Self-Efficacy (*r*=0.45; *P*=.001), and Active Coping and Seeking Support (*r*=0.42; *P*=.003), Openness to Experience and Extraversion (*r*=0.4; *P*=.005), and Extraversion and Neuroticism (*r*=0.51; *P*<.001, Extraversion and Self-Efficacy (*r*=0.51; *P*<.001). We observed moderate negative correlations between Helplessness and Active Coping (*r*=−0.56; *P*<.001), Openness to experience and Helplessness (*r*=−0.47; *P*<.001), Neuroticism and Active Coping (*r*=−0.42; *P*=.003), and Helplessness and Self-efficacy (*r*=−0.41; *P*=.004) (Figure 2).

Heatmaps with hierarchical clustering did not identify subgroups with a specific personality-functioning profile, which indicates the homogeneity of the refusing group (Figure S1 in Multimedia Appendix 1). However, if we divided the group into 4 basic refusal categories (Focus on Life Outside the Disease, Focus on Disease and Treatment, Denial Mechanism, and Technical Issues) and analyzed each psychological test separately, we observed that patients giving different reasons for refusal differed in the severity of Neuroticism, use of positive reevaluation, and Self-efficacy. Analyses showed differences in Neuroticism between the Focus on Life Outside the Disease and Focus on the disease and treatment groups (*P*=.02), with the Focus on Life Outside the Disease group showing lower levels of Neuroticism. A higher level of Positive Reinterpretation was observed in the Focus on Life Outside the Disease group than in the Technical Issues group (*P*=.05) (Tables S2 and S3 in Multimedia Appendix 1). The results showed that, compared to the Focus on Life Outside the Disease group, patients in 2 groups had lower levels of Self-efficacy: the Focus on the Disease and Treatment (*P*=.03) and Denial Reaction group (*P*=.03) (Tables S2 and S4 in Multimedia Appendix 1). When we divided the group into 3 age groups: <45, 46-60, and >60 years, the only difference was in the use of denial strategies (*P*=.004). The 46‐60 age group used this strategy more often than the ≥60 group (*P*=.04) (Tables S5 and S6 in Multimedia Appendix 1). There were no other differences in psychoemotional functioning variables between the age categories.

## Discussion

### Principal Findings

In our study, we primarily aimed to delineate the motivations and their associations with the psychoemotional functioning of patients with primary breast cancer who consciously declined the use of eHealth technology in the form of a mobile psycho-oncology app as part of a clinical trial. In line with this objective, we examined the reasons behind the refusal to participate in the clinical trial involving the app, as expressed by the patients in an open-ended question, and evaluated their psychoemotional functioning, encompassing stress levels, self-efficacy, personality traits, and coping strategies. Importantly, all psychological assessments and reasons for refusal were collected from patients who declined the app intervention as part of a clinical trial but agreed to participate in our separate observational study, indicating that their decision was app-specific rather than a general reluctance toward research involvement. In addition, we categorized patients based on their reasons for declining participation in the study and compared the psychoemotional functioning of patients within these groups.

### Refusals to Participate in a Clinical Trial Using an eHealth App

The percentage of refusals in our group is significantly higher compared to other studies [30,31]. However, there are also studies in which the refusal percentage was even higher than in our study [11]. It should be stressed that the team carrying out the clinical research on the use of the tested eHealth app did not offer participation to all consecutive patients; this could be confirmed by the upper limit of age in the tested group of patients (67 years), significantly lower than the upper age limit for patients treated by preoperative therapy in Poland. In the eligibility criteria of the trial, the skill to use the electronic tool was one of the major requirements; thus, the refusal rate is calculated in the group of patients apparently able to use it.

As mentioned previously in the Introduction section, there are limited studies that examine the reasons for rejecting the use of eHealth solutions. Gupta et al [20] listed 4 obstacles to recruiting patients for a study using a cell phone app: (1) coordination with clinic staff, (2) perceived burden among patients with breast cancer, (3) limitations on technology adoption and use, and (4) availability of resources among study staff. The reasons given by our patients corresponded to the second challenge domain identified by Gupta et al [20]. The remaining domains were less visible in our study, which may be due to a different way of constructing the refusal categories in our study compared to that of Gupta et al [20]. Among other things, when comparing these categories, we included “Unfamiliarity with mobile apps” as a “technical issue,” whereas Gupta et al [20] classified it as a “perceived burden” (2nd category). Most of the reasons for refusal cited by our patients are also commonly mentioned in other studies as reasons for withdrawal from technology-based interventions [30,31]. The only exception was “reluctance to recall disease-related content,” which we did not find in other studies. Meanwhile, patients reporting a reluctance to recall disease-related content are an interesting group from a clinical perspective, as they may be more likely to use a denial response, which is a risk factor for underestimating disease symptoms despite the available information and their intellectual capacity to comprehend it [32,33]. da Silva et al [34] demonstrated that patients with breast cancer made excuses to avoid check-ups, refrained from touching their breasts, and even avoided using the word “cancer.” It is noteworthy that in our study, patients declaring reluctance to recall content related to illness as a reason for not using the app exhibited higher levels of Neuroticism.

It would be important to consider whether reluctance to use the app was derived from reluctance to participate in the clinical trial. However, none of the patients mentioned such a reason in the open-ended question. At the same time, these patients agreed to participate in the observational study presented in this paper, which was conducted after their refusal to test the mobile app, suggesting that it was not the mere fact of “participating in the study” that was a deterrent, but the topic itself, that is, testing the phone app.

### Psychoemotional Functioning of Patients With Breast Cancer Who Declined the Use of eHealth Apps

Analysis of the results indicates that the level of stress among the patients does not appear to be the main reason for refusing to use the psycho-oncology app. Patients who declined participation may still have experienced moderate stress; however, this was not a decisive factor in their decision. This finding suggests that the refusal was more likely related to individual preferences, perceived usefulness of the app, or other psychomotor factors, rather than to low stress levels. It appears that patients who declined participation had a relatively high sense of Self-efficacy, consistent with previous Polish research [27]. It is plausible that the elevated Self-efficacy of our patients led them to forgo the use of the app, as they felt emotionally equipped to handle the challenges of illness. This conjecture aligns with Juczyński and Ogińska-Bulik’s [27] assertion that heightened Self-efficacy influences an individual’s positive assessment of personal resources in stressful circumstances. Moreover, the author contends that increased Self-efficacy is linked to engaging in health-related activities, a conclusion supported by other researchers [35,36]. However, the latter was contradicted by the results of our study, as our patients declined app usage despite possessing high Self-efficacy. This may be connected with the fact that their decision was more related to perceptions of the app itself than to a general disengagement from health-related behaviors or research. It is possible that they did not view app usage as a health-enhancing activity; this stresses the necessity of addressing this group both in the design of interventions and in the channels of their delivery at decisive moments.

In terms of coping with stress, patients most frequently used the strategies of Active Coping, Acceptance, and Seeking Emotional Support, and least frequently used the strategies of Psychoactive Substance Use and Restraint. This is a recurrent pattern of the most and least frequently used strategies in other Polish studies [37] by the authors of the test, which are considered to be highly effective in coping with stress.

Among personality dimensions, Neuroticism and Openness to Experience were relatively lower, which may help explain patients’ reluctance to use the eHealth app. Openness to Experience is conceptualized as a willingness to accept novelty in life, including new ideas and concepts [25]. Thus, the lower Openness to Experience score of our patients may indicate a lack of readiness to accept the app as something new and unfamiliar. In addition, the low Neuroticism levels of our female patients may have been a protective factor against heightened negative emotions, thereby reducing motivation to seek psychological help. It should be noted, however, that the scores on these 2 personality dimensions, although the lowest, were within Polish normative values.

### Psychoemotional Functioning in Relation to the Reasons for Refusal to Participate in the Study

Differences in the psychoemotional functioning of patients were observed based on the stated reason for refusal to participate in the study. Patients who cited focusing on life outside the disease as the reason for refusal exhibited lower levels of Neuroticism, more frequent use of positive reevaluation, and higher levels of Self-efficacy than patients giving other reasons for declining the app intervention. These psychoemotional characteristics suggest better coping with the illness and treatment situation. Lower Neuroticism is associated with a reduced tendency to experience anxiety and sadness, greater emotional balance [25], and the ability to positively reinterpret events, leading to experiencing positive emotions despite the challenges [27]. High Self-efficacy is linked to greater self-confidence and more effective action [24]. This indicates that patients who felt too focused on their life outside the disease to participate in testing the psycho-oncology app were unlikely to require additional psychological support related to a cancer diagnosis. However, 3 other groups were identified: the “Denial Reaction” group had lower levels of Self-efficacy, the group citing technical difficulties as a reason for refusal was less likely to use the positive reappraisal technique, and the group focused on the disease and treatment had lower levels of Self-efficacy and higher levels of Neuroticism. Overall, the study group tended not to exhibit significantly elevated levels of stress and tended to have high levels of Self-efficacy. The aforementioned 3 groups of patients could potentially have needed additional psycho-oncological support that the app could have provided but did not make use of it.

### Clinical Implications

Our research shows that when exploring the reasons behind patients’ reluctance to use eHealth solutions, the refusing group should not be considered uniform. Our patients provided various reasons for their refusal to use a psycho-oncology mobile app, and their psychoemotional functioning differed depending on the reason. The aforementioned 3 groups of patients could have potentially benefited from using the psycho-oncological app, but the standard procedure of inviting them to use the app (direct contact by a nurse during routine pretreatment visit) did not effectively persuade them. When implementing eHealth solutions for routine psycho-oncological support of patients with breast cancer, it is essential to understand the reasons for patients’ refusal to engage with such interventions, as this enables the use of more personalized incentives. Similar recommendations have been made for patients refusing to participate in clinical trials [38]. In addition, we recommend screening for psychoemotional functioning in patients with breast cancer before they are offered support in the form of an eHealth intervention. Such screening allows, first, the identification of patients who do not need additional support and, second, the presentation of screening results to patients as a personalized prompt (eg, indicating that the use of denial mechanisms or low self-efficacy potentially makes them more emotionally vulnerable during treatment, which they can effectively counteract with the support of the mobile app).

The most crucial issue is the problem of refusal rate in the context of eHealth interventions. Increasing the approval rate for novel tools is essential for understanding their population-level impact; possibly, the idea of carrying on the trial on both patients agreeing to use the tool and those refusing but who agreed to participate in this observational study would lead to the most reliable results of investigations. Nevertheless, we deliberately chose to focus this paper solely on the latter group. Our primary aim was to explore and categorize the reasons for refusing to use the eHealth app—a topic that, to our knowledge, has not yet been systematically addressed in the literature. Including comparative data at this stage might obscure the clarity of our categorization and shift the focus away from our core research objective. We acknowledge the importance of such a comparison and have collected data from users of the app; we intend to publish a separate article presenting a detailed comparison between users and nonusers. In addition, including these data here would significantly increase the length and complexity of this paper. We would like to draw attention again to the lack of standardized tools for investigating patients’ reasons for refusing to use psychological eHealth tools [18]. The procedure we used to categorize patients’ reasons for refusal was due to the lack of these tools. Importantly, the refusal we examined in this study pertained specifically to the use of the eHealth app, not to participation in clinical research more broadly. The results of our study, showing the different psychoemotional functioning of patients depending on the reason for refusal, indicate that this procedure was accurate and valid. We hope that this way of analyzing the reasons for patients’ refusal to use psychological eHealth tools will be developed in further studies.

Moreover, our review of the literature revealed that the research tools we used are not commonly used. However, in our opinion, they are valuable and reliable as a standard data source of diagnostic information, especially considering the relatively short time required for patients to complete them. We therefore recommend these tools for psychological screening of patients with breast cancer.

### Study Limitations

In our study, we used a small sample of a relatively homogeneous group of patients in a single breast cancer center. The generalizability to more diverse populations, patients in other geographic areas, or centers with different practices should be tested in the future. Poland is a country with a relatively homogeneous population of Caucasians, and in further studies, we will consider broader collaboration to increase diversity among participants.

In addition, although the intervention is briefly described as a psycho-oncology mobile app, we acknowledge the need for a more detailed presentation of its content, functionality, and interactivity. This information will be provided in a separate publication specifically dedicated to the intervention’s development and implementation.

We also recognize that health care infrastructure and digital health literacy vary across countries. As such, the generalizability of our findings may be limited in settings with different eHealth accessibility or patient attitudes toward digital care. While our findings may not directly generalize to countries with highly advanced digital health systems, we believe they are especially relevant for regions where psychological eHealth solutions are still emerging and face similar adoption barriers.

We acknowledge the potential impact of self-selection bias. Our participants represent a specific subset of patients who refused to use the app but agreed to participate in the observational study. Their willingness to engage in research, despite refusing the app intervention, may indicate characteristics that differentiate them from patients who declined both. This bias should be considered when interpreting the findings. A comparative analysis involving patients who used the app is currently in preparation and will be presented in a separate paper.

Despite these limitations, we believe that this study enriches our knowledge of the psychoemotional functioning of patients with breast cancer who refuse to use psychological eHealth tools in the form of a mobile app and their reasons for declining the app-based intervention, rather than research participation in general.

As a broader contextual note, the attitude of citizens in various countries to adopt novel solutions, especially in the technological domain, plays a key role. According to the latest Eurostat report [39], Poland is significantly below the European average in terms of technological expenditures and dissemination. However, numerous eServices are operational, including public health eServices, and the general enthusiasm for the use of various simple digital services (with ePayments at the forefront) is significant. For example, in 1 study designed by Finnish and Polish researchers, children were offered a mobile app intervention; Polish parents agreed in 92% of cases, while Finnish parents agreed in 56% [40], even though Finland rates significantly higher than Poland on the majority of Eurostat measures of technological enthusiasm. Seventy percent of Polish adults accept using web-based services for health-related issues [41], and this percentage is comparable to the level of Acceptance observed in our study. Thus, we consider our results potentially generalizable to a wider European population in countries with significant barriers to the adoption of psychological eHealth tools.

### Conclusions

When incorporating eHealth solutions into the regular psycho-oncological support for patients with breast cancer, it is crucial to understand the reasons behind their refusal to use such solutions. In addition, it is important to consider these reasons in the context of the patients’ psychoemotional well-being. This understanding allows for the implementation of more personalized incentives for those who could benefit from eHealth but are hesitant to use it. To achieve this, we recommend screening the patients’ psychoemotional functioning.

## Supplementary material

10.2196/71412 Multimedia Appendix 1 Additional tables and figure.

## Abbreviations

GSES: General Self-Efficacy Scale
Mini-COPE: Coping Orientation to Problems Experienced Questionnaire
TIPI: Ten-Item Personality Inventory
