# Supplementary material for: Reluctance to Use a Psycho-Oncology Mobile App Among Patients With Primary Breast Cancer: Retrospective Cross-Sectional Survey
Source: JMIR Mhealth Uhealth. 2026 Feb 13;14:e71412. doi: 10.2196/71412 (PMC12904500; doi:10.2196/71412)
Supplement: Multimedia Appendix 1 [file mhealth-v14-e71412-s001.docx]

**Table S1**. Categorization of reasons for refusal of a mobile psycho-oncology app among primary breast cancer patients in Gliwice, Poland (2022-2023), with illustrative patient statements. Data are presented as basic reasons and overarching categories.

| **Reasons of refusal** | | | |
| --- | --- | --- | --- |
| **Examples of patient responses** | **Basic reasons** |  | **Overarching categories of reasons** |
| “I have a small child, with whom I have no time for anything",  "I am too busy taking care of my children",  "I am the only person to take care of my child, I have no time for anything” | Lack of time - care of child |  | Focus on life outside the disease |
| “My mother had a stroke 2 years ago, I'm the only one who cares about her”  “My husband is disabled, he has multiple sclerosis and I have to do everything around him, I have no time for nothing else” | Lack of time - difficult family situation |  |  |
| "I'm too absorbed in my professional work",  "I have a very responsible and demanding job and have no time for anything else" | Lack of time - demanding job |  |  |
| "Hospital visits are so frequent that I no longer have time for anything" | Lack of time – frequency of medical appointments |  | Focus on the disease and treatment |
| "I had no strength for anything, I was mentally exhausted",  "I felt terrible at the beginning of treatment, I didn't have a chance to focus on anything". | Poor physical and mental well-being |  |  |
| "I didn't want to think about the disease at home either",  "Thinking about the disease was the last thing I wanted to do" | Reluctance to recall content related to the disease |  | Denial reaction |
| “I didn't feel the need for support”  “The diagnosis didn't change how I felt so I didn't see the need for a psycho-oncology application” | No mental need, feeling good |  |  |
| “I use the phone only when I have to”,  “I hate this technology everywhere”  “I couldn't imagine it, to talk to the phone, some app about how bad it was for me” | Reluctance toward new technologies |  | Technical issues |
| „I can't install anything on my work phone” | Refusal to phone contact |  |  |
| “I had been in psychotherapeutic contact for many years and I found it as a sufficient support for psycho-oncological issues as well” | Other reason |  | Other reasons |

**Figure S1**. Heatmap with hierarchical clustering of psychological test results for selected patients from the refusal group among primary breast cancer patients in Gliwice, Poland (2022-2023) (with no missing data).


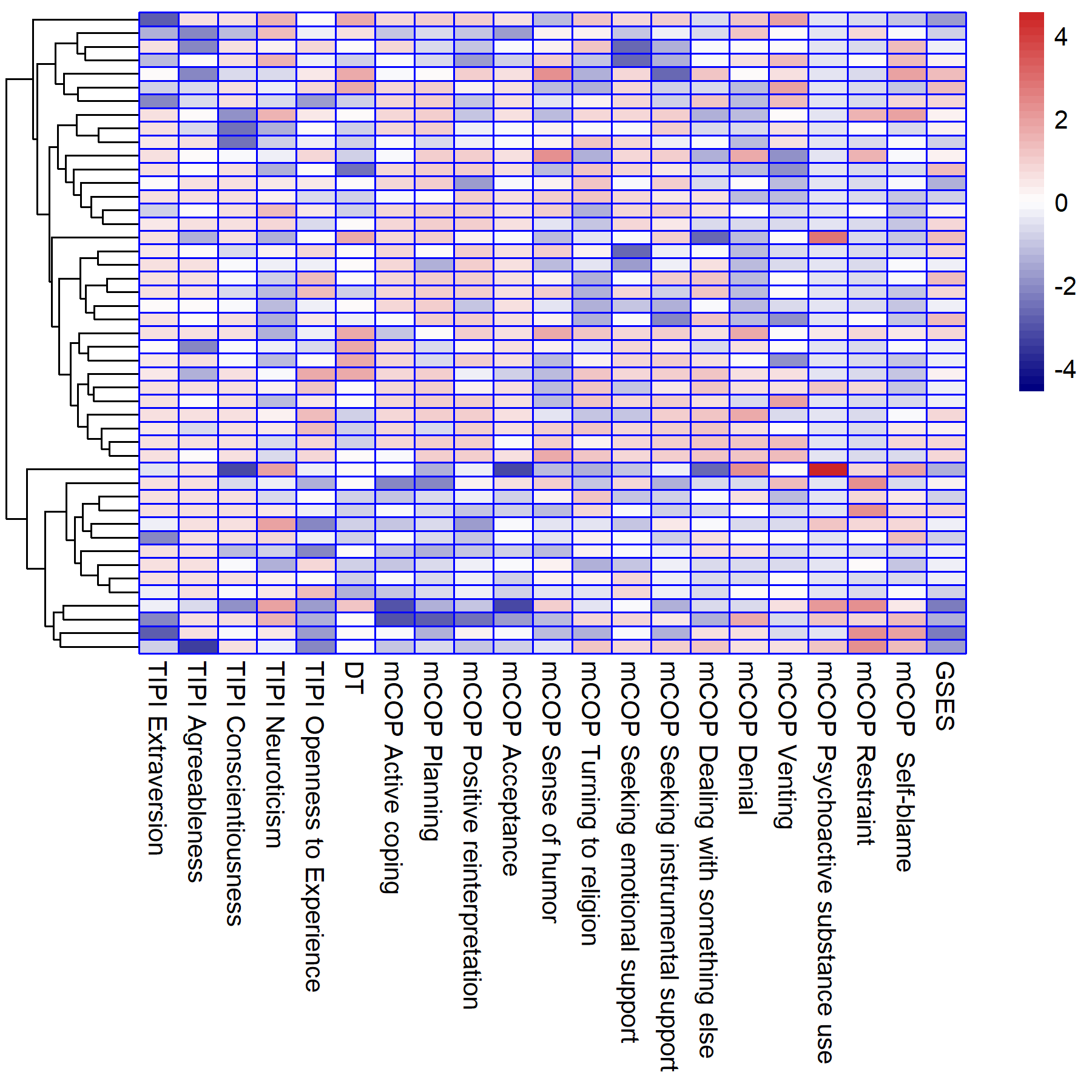


**Table S2**. Comparisons of psychology tests: stress level (DT), generalized self-efficacy (GSES), personality trait characteristic (TIPI) and stress coping strategies (Mini-Cope) between reasons of refusal to use psycho-oncological app among primary breast cancer patients in Gliwice, Poland (2022-2023).

| **Characteristic** | **Technical issues**,  n = 7 | **Focus on life outside the disease**,  n = 13 | **Focus on the disease and treatment**,  n = 20 | **Denial reaction**, n = 15 | ***P*-value**^1^,  ***(effect size calculated if P*-value** ***<.05 only)*** |
| --- | --- | --- | --- | --- | --- |
| TIPI Extraversion |  |  |  |  | .91 |
| Mean (SD) | 11.1 (3.5) | 11.8 (3.3) | 11.4 (4.0) | 12.2 (2.2) |  |
| Median (IQR) | 12.0 (9.5, 14.0) | 14.0 (9.0, 14.0) | 13.0 (11.0, 14.0) | 13.0 (10.5, 14.0) |  |
| TIPI Agreeableness |  |  |  |  | .80 |
| Mean (SD) | 12.9 (1.1) | 13.2 (1.1) | 13.1 (1.5) | 12.7 (2.1) |  |
| Median (IQR) | 13.0 (12.5, 13.5) | 13.0 (13.0, 14.0) | 14.0 (12.8, 14.0) | 14.0 (12.5, 14.0) |  |
| TIPI Conscientiousness |  |  |  |  | .86 |
| Mean (SD) | 12.6 (1.5) | 13.0 (1.3) | 12.9 (1.7) | 12.5 (2.3) |  |
| Median (IQR) | 13.0 (12.0, 13.5) | 14.0 (12.0, 14.0) | 14.0 (12.0, 14.0) | 14.0 (11.5, 14.0) |  |
| TIPI Neuroticism |  |  |  |  | **.03**  **(.09, Eta2[H], moderate)** |
| Mean (SD) | 7.3 (3.5) | 4.8 (2.6) | 8.4 (3.0) | 6.8 (4.5) |  |
| Median (IQR) | 6.0 (5.0, 9.5) | 4.0 (3.0, 6.0) | 8.0 (6.0, 9.3) | 6.0 (3.0, 10.0) |  |
| TIPI Openness to Experience |  |  |  |  | .43 |
| Mean (SD) | 9.6 (3.0) | 9.4 (2.2) | 7.9 (3.1) | 9.0 (3.2) |  |
| Median (IQR) | 10.0 (8.5, 11.5) | 9.0 (8.0, 11.0) | 8.0 (5.0, 10.3) | 9.0 (8.0, 10.5) |  |
| DT |  |  |  |  | .36 |
| Mean (SD) | 4.1 (2.0) | 4.8 (2.7) | 5.4 (1.6) | 4.9 (2.2) |  |
| Median (IQR) | 3.0 (3.0, 5.0) | 4.5 (3.0, 8.0) | 5.0 (5.0, 5.3) | 4.0 (3.0, 7.3) |  |
| mCOP Active coping |  |  |  |  | .71 |
| Mean (SD) | 2.6 (0.5) | 2.8 (0.3) | 2.5 (0.7) | 2.6 (0.4) |  |
| Median (IQR) | 2.8 (2.1, 3.0) | 3.0 (2.5, 3.0) | 3.0 (2.1, 3.0) | 2.5 (2.1, 3.0) |  |
| mCOP Planning |  |  |  |  | .07 |
| Mean (SD) | 2.7 (0.5) | 2.8 (0.4) | 2.1 (0.8) | 2.4 (0.5) |  |
| Median (IQR) | 3.0 (2.3, 3.0) | 3.0 (2.5, 3.0) | 2.0 (1.5, 3.0) | 2.0 (2.0, 3.0) |  |
| mCOP Positive reinterpretation |  |  |  |  | .04  **(**0.11, Eta2[H], moderate**)** |
| Mean (SD) | 1.9 (0.6) | 2.8 (0.5) | 2.3 (0.8) | 2.1 (0.7) |  |
| Median (IQR) | 1.8 (1.5, 2.0) | 3.0 (3.0, 3.0) | 2.5 (1.5, 3.0) | 2.0 (2.0, 2.5) |  |
| mCOP Acceptance |  |  |  |  | .17 |
| Mean (SD) | 2.7 (0.5) | 2.9 (0.2) | 2.5 (0.7) | 2.4 (0.7) |  |
| Median (IQR) | 3.0 (2.3, 3.0) | 3.0 (3.0, 3.0) | 3.0 (2.1, 3.0) | 2.5 (2.0, 3.0) |  |
| mCOP Sense of humor |  |  |  |  | .61 |
| Mean (SD) | 0.8 (0.9) | 1.1 (0.8) | 0.7 (0.7) | 0.8 (0.6) |  |
| Median (IQR) | 0.5 (0.5, 0.9) | 1.3 (0.6, 1.5) | 0.8 (0.0, 1.4) | 1.0 (0.1, 1.0) |  |
| mCOP Turning to religion |  |  |  |  | .59 |
| Mean (SD) | 1.4 (1.3) | 1.3 (1.4) | 1.9 (1.1) | 1.6 (1.3) |  |
| Median (IQR) | 1.5 (0.3, 2.4) | 1.0 (0.0, 2.8) | 2.0 (1.0, 3.0) | 1.8 (0.5, 3.0) |  |
| mCOP Seeking emotional support |  |  |  |  | .37 |
| Mean (SD) | 2.7 (0.5) | 2.9 (0.2) | 2.4 (0.7) | 2.5 (0.6) |  |
| Median (IQR) | 3.0 (2.3, 3.0) | 3.0 (2.6, 3.0) | 2.5 (2.1, 3.0) | 2.8 (2.0, 3.0) |  |
| mCOP Seeking instrumental support |  |  |  |  | .40 |
| Mean (SD) | 2.0 (0.8) | 1.9 (1.0) | 2.2 (0.8) | 2.5 (0.6) |  |
| Median (IQR) | 1.8 (1.5, 2.8) | 2.0 (1.5, 2.5) | 2.3 (1.6, 3.0) | 2.5 (2.0, 3.0) |  |
| mCOP Dealing with something else |  |  |  |  | .07 |
| Mean (SD) | 1.8 (0.8) | 2.6 (0.6) | 2.0 (0.6) | 2.1 (0.8) |  |
| Median (IQR) | 1.8 (1.1, 2.0) | 3.0 (2.5, 3.0) | 1.8 (1.5, 2.5) | 2.3 (1.6, 2.9) |  |
| mCOP Denial |  |  |  |  | .08 |
| Mean (SD) | 0.8 (1.0) | 0.5 (0.7) | 1.2 (0.8) | 1.1 (0.9) |  |
| Median (IQR) | 0.5 (0.0, 1.4) | 0.0 (0.0, 0.9) | 1.3 (0.5, 1.9) | 1.0 (0.5, 1.5) |  |
| mCOP Venting |  |  |  |  | .70 |
| Mean (SD) | 1.2 (0.9) | 1.4 (1.0) | 1.5 (0.8) | 1.6 (0.6) |  |
| Median (IQR) | 1.3 (0.6, 1.5) | 1.5 (0.8, 1.9) | 1.3 (1.0, 1.9) | 1.8 (1.1, 2.0) |  |
| mCOP Psychoactive substance use |  |  |  |  | .06 |
| Mean (SD) | 0.0 (0.0) | 0.0 (0.0) | 0.1 (0.4) | 0.5 (0.8) |  |
| Median (IQR) | 0.0 (0.0, 0.0) | 0.0 (0.0, 0.0) | 0.0 (0.0, 0.0) | 0.0 (0.0, 0.9) |  |
| mCOP Restraint |  |  |  |  | .21 |
| Mean (SD) | 0.7 (0.8) | 0.1 (0.2) | 0.6 (0.9) | 0.6 (0.6) |  |
| Median (IQR) | 0.5 (0.0, 1.4) | 0.0 (0.0, 0.0) | 0.0 (0.0, 1.0) | 0.5 (0.0, 1.0) |  |
| mCOP Self-blame |  |  |  |  | .74 |
| Mean (SD) | 1.4 (1.0) | 1.2 (1.2) | 1.1 (0.9) | 1.0 (1.1) |  |
| Median (IQR) | 1.3 (1.0, 1.9) | 0.8 (0.0, 2.4) | 1.0 (0.5, 1.5) | 0.5 (0.0, 1.9) |  |
| GSES |  |  |  |  | .02  (0.13, Eta2[H], moderate) |
| Mean (SD) | 7.1 (1.2) | 8.7 (1.5) | 6.7 (2.3) | 7.1 (1.3) |  |
| Median (IQR) | 7.0 (6.0, 8.0) | 9.0 (8.0, 10.0) | 7.0 (5.0, 9.0) | 7.0 (7.0, 8.0) |  |
| ^1^Kruskal-Wallis rank sum test | | | | | |

**Table S3**. Dunn’s test P value and P adjusted between groups- mCOP Positive reinterpretation between refusal reason categories among primary breast cancer patients in Gliwice, Poland (2022-2023).

| **mCOP Positive reinterpretation** | | |
| --- | --- | --- |
| Comparisons | *P*-value | *P-*adjusted |
| Technical issues vs  Focus on life outside the disease | 0.01 | 0.08 |
| Technical issues vs Focus on the disease and treatment | 0.27 | 0.41 |
| Focus on life outside the disease vs  Focus on the disease and treatment | 0.06 | 0.11 |
| Technical issues  vs  Denial reaction | 0.58 | 0.58 |
| Focus on life outside the disease vs  Denial reaction | 0.02 | 0.047 |
| Focus on the disease and treatment  vs  Denial reaction | 0.49 | 0.59 |

**Table S4**. Dunn’s test P value (adjusted) for generalized self-efficacy (GSES) between refusal reason categories among primary breast cancer patients in Gliwice, Poland (2022-2023).

| **GSES** | | |
| --- | --- | --- |
| Comparisons | *P* value | *P* adjusted |
| Technical issues vs  Focus on life outside the disease | .03 | .07 |
| Technical issues vs Focus on the disease and treatment | .98 | .98 |
| Focus on life outside the disease vs  Focus on the disease and treatment | .005 | **.03** |
| Technical issues  vs  Denial reaction | .96 | 1.00 |
| Focus on life outside the disease vs  Denial reaction | .01 | **0.03** |
| Focus on the disease and treatment  vs  Denial reaction | .97 | 1.00 |

**Table S5**. Comparisons of psychology tests: stress level (DT), generalized self-efficacy (GSES), personality trait characteristic (TIPI), and stress coping strategies (Mini-Cope) between age groups among primary breast cancer patients in Gliwice, Poland (2022-2023).

| **Characteristic** | **age<=45**,  n = 20 | **age 45-60**,  n = 25 | **age >=60**,  n = 11 | ***P*-value**^1^  ***(effect size calculated if P*-value** ***<.05 only)*** |
| --- | --- | --- | --- | --- |
| TIPI Extraversion |  |  |  | .96 |
| Mean (SD) | 12.1 (2.4) | 11.4 (4.1) | 11.7 (2.8) |  |
| Median (IQR) | 13.0 (10.8, 14.0) | 14.0 (9.0, 14.0) | 12.0 (11.0, 14.0) |  |
| TIPI Agreeableness |  |  |  | .76 |
| Mean (SD) | 13.1 (1.4) | 12.9 (1.6) | 12.7 (1.7) |  |
| Median (IQR) | 14.0 (12.8, 14.0) | 14.0 (13.0, 14.0) | 13.0 (12.5, 14.0) |  |
| TIPI Conscientiousness |  |  |  | .42 |
| Mean (SD) | 12.6 (1.8) | 13.1 (1.5) | 12.5 (2.1) |  |
| Median (IQR) | 13.0 (12.0, 14.0) | 14.0 (13.0, 14.0) | 13.0 (11.5, 14.0) |  |
| TIPI Neuroticism |  |  |  | .99 |
| Mean (SD) | 7.1 (4.1) | 6.7 (3.0) | 7.0 (4.5) |  |
| Median (IQR) | 6.0 (3.0, 10.0) | 7.0 (4.0, 8.0) | 6.0 (3.0, 10.5) |  |
| TIPI Openness to Experience |  |  |  | .52 |
| Mean (SD) | 8.9 (2.4) | 9.1 (3.2) | 7.8 (3.0) |  |
| Median (IQR) | 9.0 (7.8, 11.0) | 9.0 (8.0, 11.0) | 8.0 (6.0, 9.5) |  |
| DT |  |  |  | .50 |
| Mean (SD) | 5.0 (2.0) | 5.3 (2.0) | 4.3 (2.4) |  |
| Median (IQR) | 5.0 (3.0, 6.5) | 5.0 (3.0, 8.0) | 5.0 (3.0, 5.0) |  |
| mCOP Active coping |  |  |  | .82 |
| Mean (SD) | 2.6 (0.5) | 2.6 (0.5) | 2.6 (0.7) |  |
| Median (IQR) | 2.8 (2.1, 3.0) | 2.5 (2.5, 3.0) | 3.0 (2.3, 3.0) |  |
| mCOP Planning |  |  |  | .71 |
| Mean (SD) | 2.4 (0.6) | 2.4 (0.7) | 2.6 (0.6) |  |
| Median (IQR) | 2.3 (2.0, 3.0) | 2.5 (2.0, 3.0) | 3.0 (2.1, 3.0) |  |
| mCOP Positive reinterpretation |  |  |  | .96 |
| Mean (SD) | 2.3 (0.8) | 2.3 (0.7) | 2.3 (0.7) |  |
| Median (IQR) | 2.5 (2.0, 3.0) | 2.5 (1.5, 3.0) | 2.3 (1.5, 3.0) |  |
| mCOP Acceptance |  |  |  | .42 |
| Mean (SD) | 2.5 (0.7) | 2.6 (0.5) | 2.7 (0.8) |  |
| Median (IQR) | 2.8 (2.5, 3.0) | 2.5 (2.0, 3.0) | 3.0 (3.0, 3.0) |  |
| mCOP Sense of humor |  |  |  | .40 |
| Mean (SD) | 0.9 (0.5) | 0.7 (0.8) | 0.8 (0.7) |  |
| Median (IQR) | 1.0 (0.6, 1.5) | 0.5 (0.0, 1.0) | 0.5 (0.1, 1.4) |  |
| mCOP Turning to religion |  |  |  | .10 |
| Mean (SD) | 1.2 (1.1) | 2.0 (1.2) | 1.4 (1.1) |  |
| Median (IQR) | 1.0 (0.1, 2.0) | 2.5 (1.0, 3.0) | 1.0 (0.6, 2.4) |  |
| mCOP Seeking emotional support |  |  |  | .47 |
| Mean (SD) | 2.4 (0.7) | 2.7 (0.5) | 2.6 (0.6) |  |
| Median (IQR) | 2.5 (2.0, 3.0) | 3.0 (2.5, 3.0) | 3.0 (2.1, 3.0) |  |
| mCOP Seeking instrumental support |  |  |  | .42 |
| Mean (SD) | 2.1 (0.8) | 2.3 (0.9) | 2.2 (0.8) |  |
| Median (IQR) | 2.0 (1.5, 2.5) | 2.5 (2.0, 3.0) | 2.3 (1.6, 2.9) |  |
| mCOP Dealing with something else |  |  |  | .26 |
| Mean (SD) | 1.9 (0.7) | 2.3 (0.9) | 2.1 (0.7) |  |
| Median (IQR) | 1.8 (1.5, 2.4) | 2.5 (1.5, 3.0) | 2.3 (1.5, 2.5) |  |
| mCOP Denial |  |  |  | .04  (0.08, Eta2[H], moderate) |
| Mean (SD) | 0.9 (0.9) | 1.2 (0.8) | 0.5 (0.8) |  |
| Median (IQR) | 0.5 (0.1, 1.5) | 1.5 (0.5, 1.5) | 0.0 (0.0, 0.9) |  |
| mCOP Venting |  |  |  | .46 |
| Mean (SD) | 1.3 (0.9) | 1.6 (0.7) | 1.4 (0.7) |  |
| Median (IQR) | 1.3 (0.6, 1.9) | 1.5 (1.0, 2.0) | 1.5 (1.0, 1.9) |  |
| mCOP Psychoactive substance use |  |  |  | .80 |
| Mean (SD) | 0.2 (0.7) | 0.2 (0.5) | 0.2 (0.5) |  |
| Median (IQR) | 0.0 (0.0, 0.0) | 0.0 (0.0, 0.0) | 0.0 (0.0, 0.0) |  |
| mCOP Restraint |  |  |  | .89 |
| Mean (SD) | 0.4 (0.6) | 0.5 (0.8) | 0.5 (0.7) |  |
| Median (IQR) | 0.3 (0.0, 0.9) | 0.0 (0.0, 1.0) | 0.0 (0.0, 0.9) |  |
| mCOP Self-blame |  |  |  | .33 |
| Mean (SD) | 0.9 (1.0) | 1.3 (1.1) | 1.1 (0.9) |  |
| Median (IQR) | 0.5 (0.0, 1.4) | 1.0 (0.5, 2.5) | 1.0 (0.5, 1.5) |  |
| GSES |  |  |  | .71 |
| Mean (SD) | 7.5 (1.5) | 7.2 (2.2) | 7.7 (2.0) |  |
| Median (IQR) | 7.0 (6.0, 9.0) | 7.0 (6.0, 9.0) | 8.0 (7.0, 9.0) |  |
| ^1^Kruskal-Wallis rank sum test | | | | |

**Table S6**. Dunn’s test P value and P adjusted - mCOP Denial across age groups among primary breast cancer patients in Gliwice, Poland (2022-2023).

| mCOP Denial | | |
| --- | --- | --- |
| Comparisons | *P*-value | *P*-adjusted |
| age <=45  vs  age 45-60 | .17 | .26 |
| age <=45  vs  age >=60 | .19 | .19 |
| age 45-60  vs  age >=60 | .01 | .04 |
